# Spatial Variation of the Effect of Multidimensional Urbanization on PM_2.5_ Concentration in the Beijing–Tianjin–Hebei (BTH) Urban Agglomeration

**DOI:** 10.3390/ijerph182212077

**Published:** 2021-11-17

**Authors:** Qianyuan Huang, Guangdong Chen, Chao Xu, Weiyu Jiang, Meirong Su

**Affiliations:** 1 Research Center for Eco-Environmental Engineering, Dongguan University of Technology, Dongguan 523808, China; huangqianyuan2019@email.szu.edu.cn (Q.H.); chenguangdong@dgut.edu.cn (G.C.); jiangweiyu@dgut.edu.cn (W.J.); 2 School of Architecture and Urban Planning, Shenzhen University, Shenzhen 518060, China

**Keywords:** PM_2.5_ concentration, urbanization, spatial heterogeneity, geographically weighted regression model, BTH urban agglomeration

## Abstract

Atmospheric PM_2.5_ pollution has become a prominent environmental problem in China, posing considerable threat to sustainable development. The primary driver of PM_2.5_ pollution in China is urbanization, and its relationship with PM_2.5_ concentration has attracted considerable recent academic interest. However, the spatial heterogeneity of the effect of urbanization on PM_2.5_ concentration has not been fully explored. This study sought to fill this knowledge gap by focusing on the Beijing–Tianjin–Hebei (BTH) urban agglomeration. Urbanization was decomposed into economic urbanization, population urbanization, and land urbanization, and four corresponding indicators were selected. A geographically weighted regression model revealed that the impact of multidimensional urbanization on PM_2.5_ concentration varies significantly. Economically, urbanization is correlated positively and negatively with PM_2.5_ concentration in northern and southern areas, respectively. Population size showed a positive correlation with PM_2.5_ concentration in northwestern and northeastern areas. A negative correlation was found between urban land size and PM_2.5_ concentration from central to southern regions. Urban compactness is the dominant influencing factor that is correlated positively with PM_2.5_ concentration in a major part of the BTH urban agglomeration. On the basis of these findings, BTH counties were categorized with regard to local policy recommendations intended to reduce PM_2.5_ concentrations.

## 1. Introduction

In recent decades, China has achieved remarkable rapid urbanization; however, this has led to serious environmental problems [1,2]. For example, atmospheric PM_2.5_ pollution concentrations have risen to levels of public concern, given the risks posed to human physical and mental health [3,4]. Atmospheric PM_2.5_ concentrations are known to be strongly linked with the incidence of cardiovascular and respiratory diseases [5,6]. Moreover, there is a huge economic cost for society in trying to control PM_2.5_ pollution, especially in high-density population areas [7]. Recently, China has become recognized as one of the most PM_2.5_-polluted countries in the world [8], where 1.6 million fatalities annually are attributable to air pollution [9]. The fact that PM_2.5_ pollution has become the most prominent environmental problem in China, seriously hindering the process of sustainable development, has prompted considerable academic interest in the driving factors of PM_2.5_ pollution.

PM_2.5_ pollution is determined by two important influencing factors, namely, PM_2.5_ emission and purification. On the one hand, the factors that lead to PM_2.5_ emissions are extremely complex. In addition to the emission of particle pollutants, other factors that are related to urbanization, such as traffic transportation (e.g., traffic congestion, public transport provision, and road construction), industrial production (e.g., petrochemical industry, construction industry, and mining industry), residential consumption (e.g., incense burning, domestic cooking, and heating), and so on, have also played an important role in affecting PM_2.5_ emission [10,11,12,13,14]. On the other hand, the air purification capacity could effectively mitigate PM_2.5_ pollution, which is jointly determined by the air dispersion conditions and the air cleaning services of the ecosystem. First, the air dispersion conditions are mainly affected by meteorological conditions, which have been widely discussed in relevant studies, e.g., [3,15,16]. Second, air cleaning services provided by the ecosystem are largely determined by the scale, distribution, and structure of ecological space, which has been greatly influenced by the process of rapid urbanization. Considering the complexity of the driving mechanism of PM_2.5_ pollution, it is difficult to include all factors in one research. Accordingly, existing studies mainly tend to focus on one or a few groups of factors to investigate their impact on PM_2.5_ concentrations. For example, Shen et al. explored the influence of urbanization-induced population migration on ambient PM_2.5_ concentrations in China and found a reduction in PM_2.5_ exposure due to migration [17].

Urbanization is considered to be the primary driver of China’s PM_2.5_ pollution [18]. On the one hand, economic growth associated with urbanization is accompanied by massive energy consumption, increased industrial pollutant emissions, and heightened traffic volumes, which lead directly to increased PM_2.5_ emissions [19,20]. On the other hand, urbanization has led to major expansion of the area of impervious surfaces and encroachment on forests, grasslands, and other ecological lands, leading to decline in ecosystem services and reduction in PM_2.5_ purification capacity [21]. Therefore, establishing how best to achieve sustainable urbanization development in China represents a major challenge.

With the aim of reducing atmospheric PM_2.5_ concentrations, many previous studies focused on investigating the relationship between urbanization and PM_2.5_ pollution. For example, numerous studies have shown that urbanization has significant influence on PM_2.5_ concentrations at different levels [22,23]. Using a panel model, Luo et al. established that PM_2.5_ concentration has a positive relationship with urbanization in the Beijing–Tianjin–Hebei (BTH) region in China [24]. Wang et al. found that high levels of urban land size, population, share of secondary industry, and population density have increased PM_2.5_ concentrations in Chinese cities [25]. However, most related studies regarded their specific study area as a homogeneous unit with little consideration of the heterogeneity of cities. The heterogeneity in the association between PM_2.5_ pollution and urbanization means that urbanization might exert different effects on PM_2.5_ concentration in cities in different locations, given that cities differ in terms of their economic development and natural conditions [26]. Therefore, assessment of such spatial heterogeneity is conducive to developing deeper understanding of this association.

The question of how best to measure urbanization is of major importance in related studies. Often, a single indicator is used to measure urbanization, e.g., gross domestic product (GDP) density [3] and proportion of urban inhabitants to the total population [27]. However, urbanization is a complex process of transformation in cities, which is accompanied by a series of phenomena such as economic growth, population agglomeration, lifestyle changes, and technological progress [28,29]. Therefore, some recent studies have attempted to characterize urbanization from multiple dimensions, and the most popular classification approach is to divide urbanization into land urbanization, economic urbanization, and population urbanization [15,30].

In general, the spatial heterogeneity of the association between urbanization and PM_2.5_ concentration is somehow overlooked in current research studies. Set against this backdrop, the purpose of the present study is to fill this research gap by adopting the BTH urban agglomeration as the study area. To this end, urbanization was assessed from three dimensions, i.e., economic, population, and land urbanization, to comprehensively explore its relationship with PM_2.5_ concentrations. Then, the heterogeneity of the association was investigated at the county level using a geographically weighted regression (GWR) model. Finally, by grouping the influence effect, policy recommendations aimed at the reduction of PM_2.5_ concentrations were proposed for each group of counties.

## 2. Study Area

**Figure 1:**
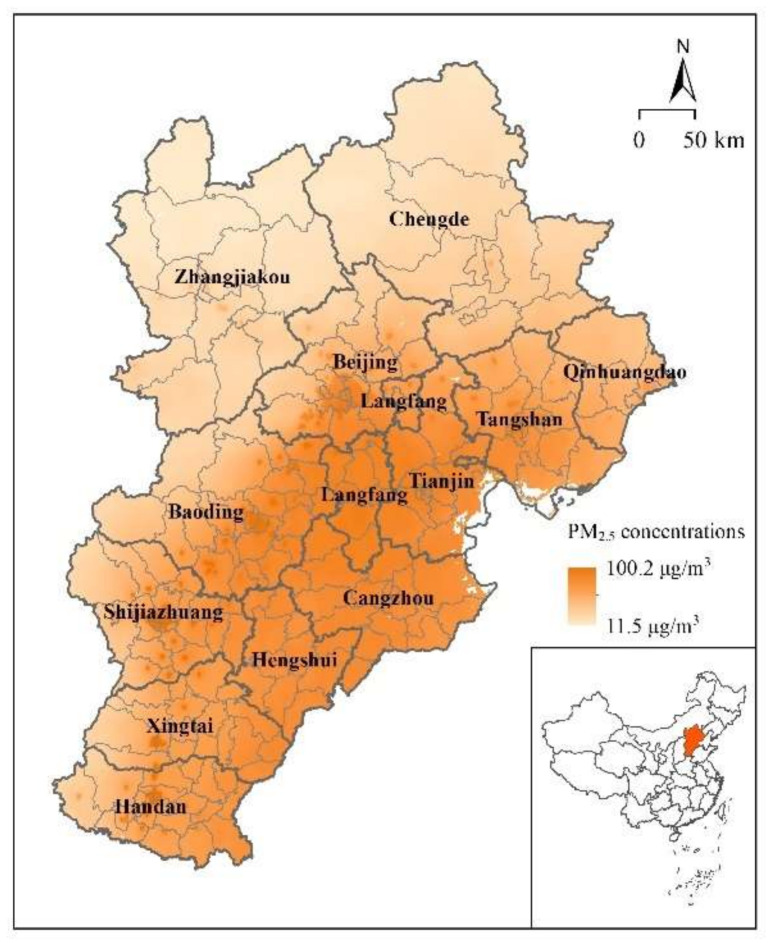
Location of the Beijing–Tianjin–Hebei urban agglomeration and the distribution of atmospheric PM_2.5_ concentration in 2015.

The BTH urban agglomeration, located in the north of China, comprises 2 municipalities (Beijing and Tianjin) and 11 prefecture-level cities (Chengde, Zhangjiakou, Langfang, Tangshan, Qinhuangdao, Baoding, Shijiazhuang, Hengshui, Cangzhou, Xingtai, and Handan; Figure 1). It is one of the most economically developed regions in China, supporting 8.4% of the national population and generating 10.24% of the country’s total GDP in 2015 [15]. With the remarkable urbanization that has occurred in recent decades, the urban area of the BTH region has nearly doubled between 1990 and 2015, leading to massive loss of farmland, grassland, and forest, which in turn has resulted in rapid reduction of ecosystem services [31]. Under this circumstance, many environmental problems have become increasingly prominent, especially the level of atmospheric PM_2.5_ pollution, which has raised widespread concern. A report by the Ministry of Environmental Protection in 2015 noted that 7 of the 10 cities with the worst air quality in China were within the BTH urban agglomeration, and that the BTH region was the area with the worst air quality in the country [32]. Consequently, reduction of atmospheric PM_2.5_ concentration within the BTH region is highly urgent.

## 3. Data and Methods

### 3.1. Selection of Urbanization Indicators and Data Sources

Urbanization brings complex and comprehensive change to cities, mainly in terms of the population, economy, and land [28]. Urbanization contributes to massive population growth and population aggregation [33], promoting concentration of economic activity [34]. Rapid growth of an urban population requires more land to host the associated socioeconomic activities, leading to dramatic transformation of urban land. In the context of rapid urbanization, the area of urban land expands continuously and shows trends of densification and compactness [35,36]. In the view of the above, this study adopted total GDP and population (POP) to represent economic urbanization and population urbanization, respectively. With regard to land urbanization, urban land size has been commonly used as an indicator to measure land urbanization, usually measured by the extent of the built-up area [15]. However, as another important characteristic of land urbanization, urban compactness has also been shown to have a significant influence on PM_2.5_ concentrations [37]. Accordingly, two commonly used landscape indexes, i.e., the largest patch index (LPI) and percentage of like adjacencies (PLADJ), were employed to represent urban land size and compactness, which are considered to be the two main characteristics of land urbanization [37]. The LPI indicates the degree of dominance of the urban area within the landscape, while the PLADJ measures the aggregation of the urban class [38]. The LPI and PLADJ were both calculated on the basis of a land use/land cover (LULC) dataset.

Accurate measurement of PM_2.5_ concentrations is crucial for investigating the impact of urban form on PM_2.5_ concentrations [39]. The stationary monitoring data has advantages in accuracy but is less applicable for large-scale spatially explicit research. In this case, thanks to the development of high accuracy retrieval algorithms in recent years, satellite-derived PM_2.5_ grid data with long-term stability and high resolution has been increasingly widely applied [40]. In this study, a widely used satellite-derived PM_2.5_ gridded dataset was employed, which estimates global PM_2.5_ concentrations on the basis of multiple information sources by adopting the GWR model [40]. The raster datasets of GDP, POP, and LULC as well as the county boundary vector data were obtained from the Data Center for Resources and Environmental Sciences of the Chinese Academy of Sciences (RESDC). Before further analysis, all these datasets were transformed into the same projection coordinate system (WGS1984 UTM Zone 50N) and spatial resolution (30 m × 30 m) to avoid potential interference with the results (Table 1). The research was conducted for the year 2015, and the data for all variables were aggregated to the county level for further analysis. As there are 199 counties in the BTH urban agglomeration, the values of all variables for each county can be found in Supplementary Table S1.

### 3.2. Spatial Correlation Test

To investigate whether PM_2.5_ concentrations in the BTH region are spatially clustered and if so, the spatial correlation of PM_2.5_ concentrations at the county level was measured using the global Moran’s I and local indicators of spatial association (LISA), which are formulated as follows [32]:

(1) $$Moran’s\;I=\frac{\sum_{i=1}^{n}\sum_{j=1}^{n}w_{ij}(x_{i}-\bar{x})(x_{j}-\bar{x})}{\left(\sum_{i=1}^{n}\sum_{j=1}^{n}w_{ij}\right)\sum_{i=1}^{n}{\left(x_{i}-\bar{x}\right)}^{2}},\;\left(i\neq j\right)$$

where $x_{i}$ and $x_{j}$ denote the PM_2.5_ concentration of the *i*-th and *j*-th county in 2015, respectively; $\bar{x}$ denotes the average PM_2.5_ concentration among all counties; n is the total number of counties in the BTH urban agglomeration; and $w_{ij}$ is the spatial weighted matrix. The value of the Moran’s I is within the range of −1 to +1; the larger its absolute value, the stronger is the degree of spatial correlation.

(2) $$Local\;Moran’s\;I=\frac{n(x_{i}-\bar{x})\sum_{j=1}^{m}w_{ij}\left(x_{j}-\bar{x}\right)}{\sum_{i=1}^{n}{\left(x_{j}-\bar{x}\right)}^{2}},\;(i\neq j)$$

where the representation of the parameters is similar to that of Equation (1), except that m is the number of counties adjacent to the *i*-th county. Normally, the result of LISA classifies counties within the study area into three categories: high–high (H-H) clustering, low–low (L-L) clustering, and high–low (H-L) clustering.

### 3.3. Geographically Weighted Regression Model

In studies related to the association between urbanization and PM_2.5_ concentration, traditional regression models such as ordinary least squares (OLS) linear regression models and panel models incorporate the common assumption that the association between the two is spatially consistent. In other words, the association does not change with spatial location [41]. However, the differing natural and socioeconomic conditions between different spatial positions lead to the existence of spatial heterogeneity [29]. It means that the association between urbanization and PM_2.5_ concentration is unlikely to be spatially homogeneous, and therefore discussion of the importance of spatial heterogeneity is necessary. In this study, the GWR model was employed because it takes full consideration of the spatial heterogeneity of the study area and incorporates location information into the regression parameter estimation [42,43]. Thus, GWR can be used to calculate local regression coefficients for each sample as follows:

(3) $$y_{i}=\beta_{0}\left(\mu_{i},\nu_{i}\right)+\sum_{k}\beta_{k}\left(\mu_{i},\nu_{i}\right)x_{k,i}+\varepsilon_{i}$$

where $y_{i}$ denotes the PM_2.5_ concentration of the *i*-th county, $x_{k,i}$ denotes the *k*-th independent variable of the *i*-th county, $\beta_{0}$ denotes the intercept term, $\beta_{k}$ is the regression parameter of the *k*-th independent variable, $\varepsilon_{i}$ is the random error term, and $\left(\mu_{i},\nu_{i}\right)$ are the coordinates of the *i*-th county. The estimation of the local regression parameter $\beta_{k}\left(\mu_{i},\nu_{i}\right)$ is formulated as follows:

(4) $${\hat{\beta }}_{k}\left(\mu_{i},\nu_{i}\right)={\left[X^{T}W\left(\mu_{i},\nu_{i}\right)X\right]}^{-1}X^{T}W\left(\mu_{i},\nu_{i}\right)y_{i}$$

where $W\left(\mu_{i},\nu_{i}\right)$ is the spatial weighted matrix of the *i*-th county. The application of the spatial function has substantial influence on the GWR results. In this study, to build the spatial matrix, we adopted the Gaussian distance decay-based function, which is a function used widely in the definition of space relation in GWR models. The formula is expressed as follows:

(5) $$W_{ij}=exp\left(-\frac{d_{ij}{}^{2}}{h^{2}}\right)$$

where $d_{ij}$ is the Euclidean distance between the *i*-th county and the *j*-th county, and h represents the bandwidth, which controls the degree of distance decay. Here, we used the method of minimizing the Akaike information criterion (AIC) to determine the optimal bandwidth. The natural logarithmic transformation was applied to all the variables to reduce the heteroscedasticity of the original data [15]. Before conducting the GWR model, the OLS regression model was applied to explore the relationship between independent variables and dependent variables. In the OLS model, a positive regression coefficient indicates a positive association between the dependent and independent variables, and vice versa.

## 4. Results and Discussion

### 4.1. Spatial Pattern of PM_2.5_ Concentration

**Figure 2:**
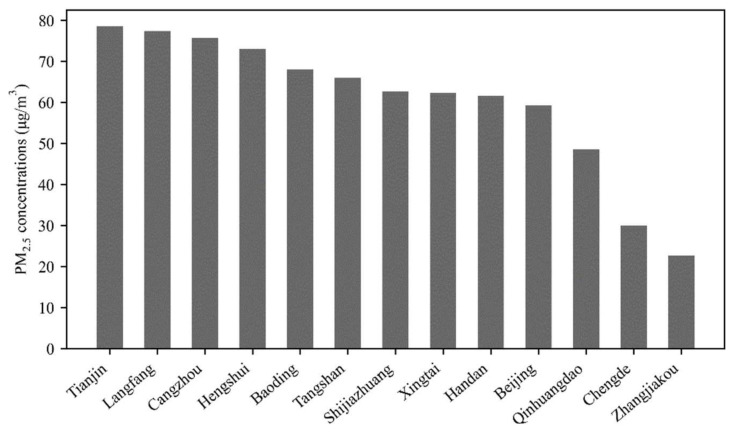
Annual average PM_2.5_ concentration of cities in the Beijing–Tianjin–Hebei urban agglomeration in 2015.

The levels of PM_2.5_ concentration in 2015 are illustrated in Figure 2. Tianjin has the highest PM_2.5_ concentration (78.55 μg/m^3^) and Zhangjiakou has the lowest PM_2.5_ concentration (22.70 μg/m^3^). Thus, the PM_2.5_ concentration in Tianjin is 3.46 times higher than that in Zhangjiakou, which illustrates the major heterogeneity of the PM_2.5_ distribution in the BTH urban agglomeration. The average annual PM_2.5_ concentration in the BTH region is 60.43 μg/m^3^. According to the Ambient Air Quality Standard posted by the Ministry of Ecology and Environment of China, the annual average PM_2.5_ concentration in cities should meet the standard of 35 μg/m^3^ to achieve good air quality. However, in 2015, only Chengde and Zhangjiakou met this requirement, while the PM_2.5_ concentration of the remaining 11 cities was substantially higher than the standard.

**Figure 3:**
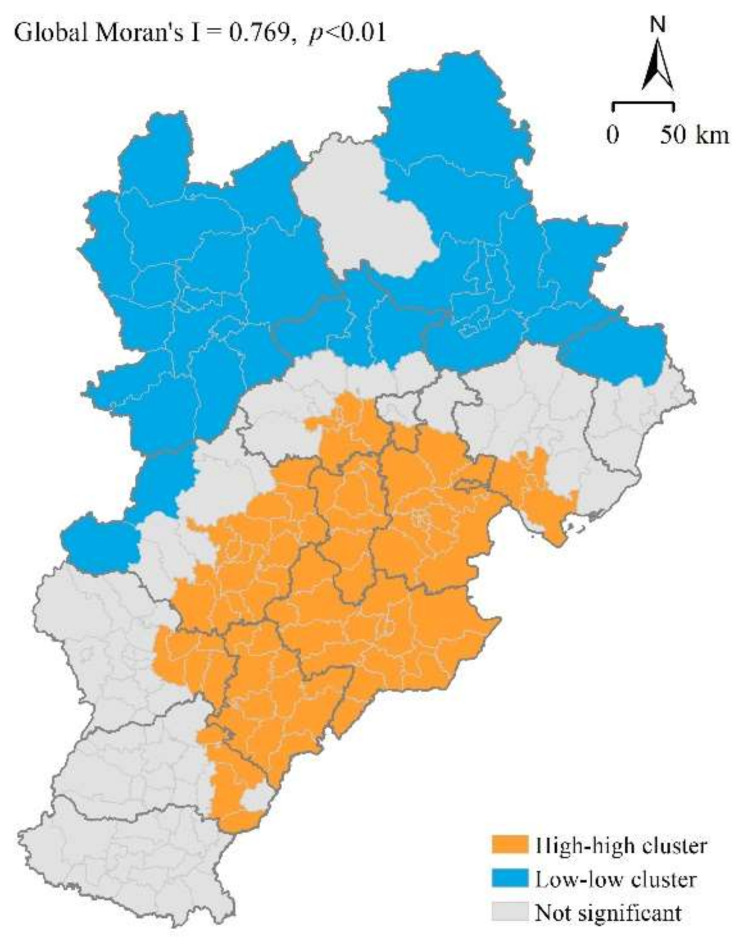
Local indicators of spatial association of PM_2.5_ concentrations in the Beijing–Tianjin–Hebei urban agglomeration in 2015.

The uneven distribution of PM_2.5_ concentration is confirmed in Figure 1, which shows that the PM_2.5_ concentration in 2015 increased from the northwest toward the southeast. This might be attributable to the large amount of vegetation distributed within the northwest region, which has greater capability for purifying air pollution [31]. To measure the clustering characteristics within the BTH urban agglomeration, spatial correlation tests were conducted. The value of the global Moran’s I was 0.769 (significant at the 1% level), indicating a significant spatial autocorrelation of the PM_2.5_ distribution. Moreover, the result of LISA confirmed a significant L-L (H-H) cluster in the northwestern (southeastern) region of the BTH urban agglomeration (Figure 3).

The clustering characteristics reflect the spatial spillover effect of PM_2.5_ pollution, which means that local PM_2.5_ pollution can have a positive impact on adjacent areas [44]. There are two possible reasons for this effect. On the one hand, PM_2.5_ pollution can spread easily from cities with high PM_2.5_ concentrations to surrounding areas because of the local atmospheric circulation [3]. On the other hand, strengthened socioeconomic connections between neighboring cities can lead to PM_2.5_ pollution associated with industrialization of a certain city affecting the level of pollution in adjacent cities [45]. The existence of the spatial spillover effect highlights the complexity of PM_2.5_ pollution regulation. It is evident that regulation is required not just regarding specific measures for pollution reduction for each city but also regarding the development of a regional policy for control of PM_2.5_ concentrations.

### 4.2. Global Regression Analysis

For the purposes of investigating the global association between urbanization and PM_2.5_ concentration and of drawing a comparison with the GWR model results, OLS regression was conducted, and the results are presented in Table 2. The variance inflation factor (VIF) of each variable was <10, indicating no significant multicollinearity among the independent variables [46]. All the regression coefficients of the independent variables were significant to at least the 10% level. The results show that GDP and LPI have a negative impact on PM_2.5_ concentrations, while POP and PLADJ have a positive effect. It means that economy and urban land size show a negative correlation with PM_2.5_ concentrations, while population and urban compactness show a positive one.

The possible reasons for this association between multidimensional urbanization and PM_2.5_ concentration are as follows. First, according to Li and Zhang, in the BTH urban agglomeration, heavy industries with high levels of pollution have been transferred from the developed counties to the less developed counties that are not urban centers. Accordingly, the levels of air pollutant emission are relatively higher in these less developed counties [47]. Conversely, urban centers tend to have a higher level of GDP and larger urban size, thus leading to a negative correlation between GDP and PM_2.5_ concentration and between the LPI and PM_2.5_ concentration. Second, dense urban populations can lead to higher energy consumption and greater traffic congestion problems, resulting in higher levels of air pollution [48]. For example, in the city of Beijing, the most densely populated city in the BTH urban agglomeration, the average time of congestion on weekdays in the first half of 2012 was 70 min [49]. Besides, the energy consumption had been increased by 191.33 million tce in Beijing over the period of 2009–2018 due to population growth. Third, urban compactness has a positive relationship with PM_2.5_, as also found in previous studies [50,51]. Although a compact urban form is proven to reduce transportation pollutant emissions by reducing commuting distances [52], in developed areas such as the BTH region in China, urban expansion has been extremely rapid and the area of green spaces has declined drastically. Thus, a compact urban form would further reduce the mix of urban and green spaces, which is not conducive to purification of air pollution [50].

A comparison of the statistical results of the fitting effect between OLS and GWR models is presented in Table 3. In terms of both R^2^ and adjusted R^2^, the values for GWR are approximately twice as large as those for OLS regression, while in terms of the value of the AIC, the result for GWR are notably smaller than that for OLS regression, suggesting that GWR is much more effective than the OLS regression model in relation to the dataset used in this research. This result further confirms the importance of incorporating location information in the regression model. Accordingly, different PM_2.5_ pollution control measures must be established in consideration of distinguish situations of different counties.

### 4.3. GWR Analysis

#### 4.3.1. Spatial Correlation between Urbanization and PM_2.5_ Concentration

**Figure 4:**
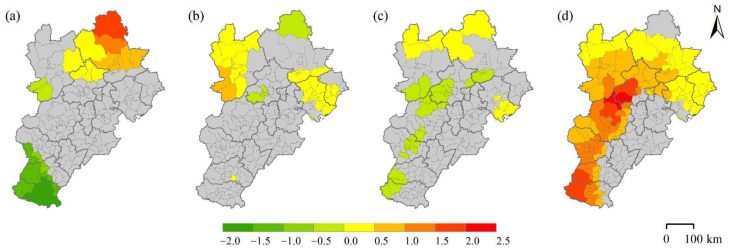
Spatial distribution of the local regression coefficients of the independent variable: (**a**) ln GDP, (**b**) ln POP, (**c**) ln LPI, and (**d**) ln PLADJ.

To estimate the degree of heterogeneity in the relationship between multidimensional urbanization and PM_2.5_ concentration in the BTH urban agglomeration, the GWR model was implemented (as described in Section 3.3) for further analysis. The estimated regression coefficients of the independent variables, illustrated in Figure 4, show that the relationship between each dimensional indicator of urbanization and PM_2.5_ concentration shows significant spatial differences. This demonstrates the complexity of the relationship, highlighting the fact that policies dedicated to improving local PM_2.5_ pollution should be tailored to the particular local context.

It can be seen from Figure 4a that GDP has a positive impact on PM_2.5_ concentrations in the northern part of the region, but a negative impact in the south. The northern part of the BTH urban agglomeration is mostly mountainous and hilly [26]. In counties with lower GDP of this region, the level of natural vegetation coverage is remarkably high, which is usually associated with better air quality. Conversely, as the counties with higher GDP are predominantly located in the urban centers, the rapid expansion of impervious surfaces in the urban centers due to rapid urban economic development has encroached on existing green spaces, resulting in relatively poor air quality. Thus, GDP has a positive effect on PM_2.5_ concentration in the northern region. In the southern region, mostly in the cities of Xingtai and Handan, the terrain is flatter and more land is available for construction. The counties surrounding the urban centers of Xingtai and Handan have a large number of coal-fired industrial enterprises, which result in serious air pollution [53]. Given this circumstance, GDP shows a negative association with PM_2.5_ concentration in the southern region.

The association between POP and PM_2.5_ concentration is relatively weak, as is the case for the association between the LPI and PM_2.5_ concentration (Figure 4b,c). Specifically, POP has a weak positive effect on PM_2.5_ concentration in northeastern and northwestern regions, whereas the LPI has a weak negative effect on PM_2.5_ concentration in some central to southern areas and a positive effect in a small part of the north. Northwestern and northeastern parts of the BTH, mainly consisting of Zhangjiakou and Qinhuangdao, respectively, have a more developed tourism sector and relatively few highly polluting industries. In this context, population size becomes the main driver of PM_2.5_ concentration. The counties in which the correlation between the LPI and PM_2.5_ concentration is negative are mainly located around better developed counties. The presence of some highly polluting industries leads to higher PM_2.5_ concentrations in these counties.

Among the four indicators, PLADJ shows the strongest and spatially widest positive relationship with PM_2.5_ concentration, demonstrating that urban compactness is the most dominant driver of PM_2.5_ pollution in multidimensional urbanization (Figure 4d). Especially in the area from Beijing to Handan, the over-compact urban form generates very high levels of PM_2.5_ pollution. There are three possible explanations for the positive relationship between urban compactness and PM_2.5_ concentration. First, compact urban development has been demonstrated to exacerbate the problem of urban traffic congestion in China, which would significantly increase PM_2.5_ emissions [35]. Second, a compact urban form would accommodate a large number of human activities in a limited space, resulting in high energy consumption and high pollution emissions [54]. Third, as a main characteristic of compact cities, land densification would lead to the reduction and uneven distribution of urban green spaces, which would further lower the capacity of air purification of the urban ecosystem [36].

#### 4.3.2. Classification of Counties and Policy Recommendations for PM_2.5_ Reduction

**Figure 5:**
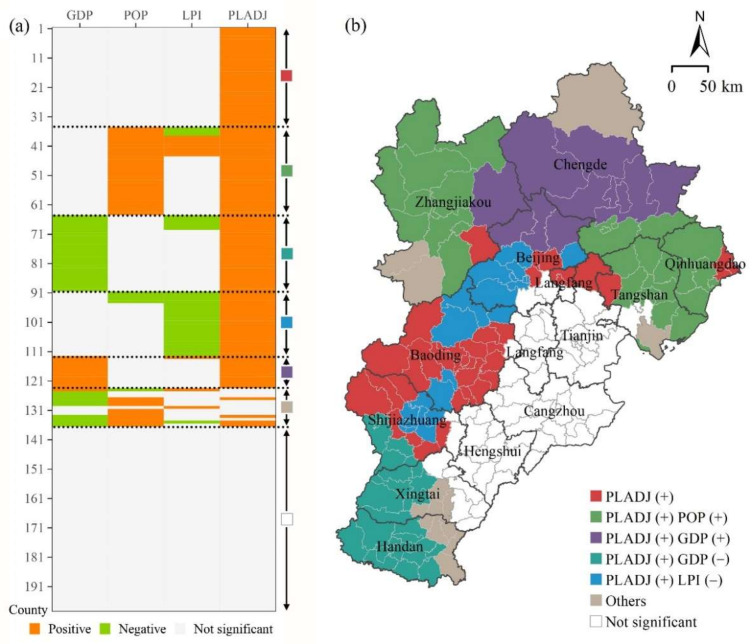
(**a**) Relationship between urbanization indicators and PM_2.5_ concentration and (**b**) associated classification of counties in the Beijing–Tianjin–Hebei urban agglomeration. The “+” and “−” symbols represent positive and negative correlation with PM_2.5_ concentration, respectively.

To develop location-specific PM_2.5_ reduction policies, all the counties within the BTH urban agglomeration were categorized into different groups based on the relationship between multidimensional urbanization and the PM_2.5_ concentration, among which five groups showed significant characteristics (Figure 5).

The first group, characterized by a positive correlation between urban compactness and PM_2.5_ concentration, comprised counties located mainly in Baoding, Shijiazhuang, Beijing, and Langfang (marked in red in Figure 5b). These counties have the most significant impact on PM_2.5_ concentration in terms of compact urban form, highlighting the priority of addressing the issue of urban over-compactness. Therefore, we suggest that counties in this group increase urban green spaces to reduce urban compactness. Meanwhile, a polycentric development pattern would be more conducive to PM_2.5_ reduction in these counties.

The second group includes counties distributed mainly in Tangshan, Qinhuangdao, and Zhangjiakou (marked in green in Figure 5b). In addition to urban compactness, population size also has a positive effect on PM_2.5_ concentration in these counties, implying that increased residential energy consumption and heightened traffic volumes generated by population concentration have significant impact on PM_2.5_ pollution [48]. In addition to adopting measures to increase green spaces, as recommended in relation to the first group of counties, these counties should also control excessive population concentration and enhance public transportation services to mitigate the associated negative effects.

The counties of the third group are gathered at the border of Chengde, Zhangjiakou, and Beijing (marked in purple in Figure 5b), and they exhibit a positive correlation between urban compactness and PM_2.5_ concentration, and between the economy and PM_2.5_ concentration. Counties in this group have a high level of vegetation coverage, which provides excellent PM_2.5_ purification capacity, especially in the underdeveloped areas. In this case, the economically developed counties in this area have a high level of PM_2.5_ concentration. Therefore, these counties should improve energy efficiency and reduce energy consumption per unit GDP, thereby mitigating the air pollution problems caused by economic development.

The counties of the fourth group are mainly located in Shijiazhuang, Xingtai, and Handan (marked in dark cyan in Figure 5b). This group is characterized by a positive correlation between urban compactness and PM_2.5_ concentration and, in contrast to the third group, a negative correlation between the economy and PM_2.5_ concentration. As mentioned in Section 4.1, such characteristics are likely due to the large number of highly polluting industries located in counties that are not regional urban centers. Counties of this group should change their energy consumption structure and reduce their use of energy sources associated with high levels of polluting emissions, such as coal.

The counties of the fifth group are distributed in Beijing, Baoding, and Shijiazhuang (marked in dark sky blue in Figure 5b). Similar to the first four categories, urban compactness shows a positive correlation with PM_2.5_ concentration, but with the difference that urban land size shows a negative correlation with PM_2.5_ concentration. This means that the pollution in these areas comes mainly from underdeveloped counties with smaller urban land size, and therefore these counties should be the focus of PM_2.5_ pollution control. Moreover, the southeastern region, mainly comprising the cities of Hengshui, Cangzhou, Tianjin, and Langfang, shows no significant correlation between urbanization and PM_2.5_ concentration. Consequently, the driver of PM_2.5_ pollution in this area might be more complex and it will require further investigation in future studies.

## 5. Conclusions and Limitations

The BTH urban agglomeration has the highest level of atmospheric PM_2.5_ pollution in China, which causes huge socioeconomic losses. The urbanization process, which has been the dominant driver of socioeconomic development in China in recent decades, has highly complex and multidimensional characteristics. It is of great importance to explore the effect of multidimensional urbanization on PM_2.5_ pollution, especially in relation to a heavily polluted region such as the BTH urban agglomeration. In this study, we employed GDP, POP, LPI, and PLADJ to measure the county-level economy, population, urban land size, and urban compactness of the BTH region. Additionally, the GWR model was used to investigate the spatial heterogeneity in the relationship between multidimensional urbanization and PM_2.5_ concentration. The results showed that the four indicators influence PM_2.5_ pollution in different ways. Economic urbanization was found to exert positive and negative effects on PM_2.5_ concentration in the northern and southern parts of the region, respectively. Population size was found slightly positively correlated with PM_2.5_ concentration in northwestern and northeastern areas. Urban land size had a negative effect on PM_2.5_ concentration from central to southern regions of the BTH. Moreover, urban compactness had the strongest positive impact on PM_2.5_ concentration, indicating that counties within the BTH urban agglomeration generally face the problem of an over-compact urban form. On the basis of the derived associations, the counties of the BTH urban agglomeration were categorized into different groups such that local policy recommendations could be properly tailored to the specific characteristics of each group of counties.

This study shed new light on the heterogeneity of the relationship between urbanization and PM_2.5_ concentration. Moreover, the findings of this study proved useful for developing PM_2.5_ reduction policies and promoting sustainable urban development within the BTH urban agglomeration. However, certain limitations of the study should be addressed in future studies. First, the driving mechanism of PM_2.5_ pollution is complicated, and there are certainly some other influencing factors such as PM_2.5_ emission, meteorological parameters, etc. However, for large-scale studies, some factors (e.g., the accurate monitoring data of PM_2.5_ emission) are difficult to acquire due to the lack of data availability. In addition, incorporating many factors into the regression model might make it difficult to meet the required minimum sample size and weaken the influences of target factors on PM_2.5_ concentrations in the analysis results. Accordingly, the main focus of this study has been placed on the influence of urbanization. Nonetheless, the other factors that could have an impact on PM_2.5_ pollution should be investigated where feasible. Second, in this study, the positive relationship between urban compactness and PM_2.5_ concentration is not based on the analysis of industrial activity or traffic volume data due to the lack of data availability. Although this argument is supported by the results of a number of recent studies, it should be further verified when more data are available. Third, our findings showed that the southeastern region, comprising mainly Tianjin, Langfang, Cangzhou, and Hengshui, has no significant correlation between urbanization and PM_2.5_ concentration. This suggests that the drivers of PM_2.5_ pollution in this region might be related to factors not specifically linked with urbanization and should be explored further in future work.

## Figures and Tables

**Table 1:** Data sources and descriptions.

Name	Type of Data	Data Sources	Spatial Resolution	Coordinate System
PM_2.5_	Raster data	Socioeconomic Data and Applications Center of Columbia University	30 m × 30 m	WGS1984 UTM Zone 50N
GPD	Raster data	RESDC	30 m × 30 m	WGS1984 UTM Zone 50N
POP	Raster data	RESDC	30 m × 30 m	WGS1984 UTM Zone 50N
LULC	Raster data	RESDC	30 m × 30 m	WGS1984 UTM Zone 50N
County boundary	Vector data	RESDC		WGS1984 UTM Zone 50N

**Table 2:** The results of the OLS regression and the VIF values for all independent variables.

	Regression Coefficient	VIF
Intercept	−0.037 **	
ln GDP	−0.259 *	3.226
ln POP	0.327 ***	2.992
ln LPI	−0.171 ***	1.930
ln PLADJ	1.011 ***	1.857
Adjusted R^2^	0.426	

Note: ***, **, and * denote *p* < 0.01, *p* < 0.05, and *p* < 0.1, respectively; VIF: variance inflation factor.

**Table 3:** Statistical test comparison of ordinary least squares (OLS) regression and geographically weighted regression (GWR).

	OLS	GWR
R^2^	0.441	0.919
Adjusted R^2^	0.426	0.886
AIC	163.641	−141.907

Note: R^2^: coefficient of determination; AIC: Akaike information criterion.

## Data Availability

The dataset used in this study was obtained from the Data Center for Resources and Environmental Sciences of the Chinese Academy of Sciences (https://www.resdc.cn/) (accessed on 30 August 2021).

## Supplementary Materials

The following are available online at https://www.mdpi.com/article/10.3390/ijerph182212077/s1, Table S1: Values of all variables for each county in the BTH urban agglomeration.

## Abbreviations

BTH	Beijing–Tianjin–Hebei
GDP	Gross domestic product
GWR	Geographically weighted regression
POP	Population
LPI	Largest patch index
PLADJ	Percentage of like adjacencies
LULC	Land use/land cover
RESDC	Data Center for Resources and Environmental Sciences of the Chinese Academy of Sciences
LISA	Local indicators of spatial association
OLS	Ordinary least squares
AIC	Akaike information criterion
VIF	Variance inflation factor
