# Supplementary material for: Spatial Variation of the Effect of Multidimensional Urbanization on PM2.5 Concentration in the Beijing–Tianjin–Hebei (BTH) Urban Agglomeration
Source: Int J Environ Res Public Health. 2021 Nov 17;18(22):12077. doi: 10.3390/ijerph182212077 (PMC8624147; doi:10.3390/ijerph182212077)
Supplement: Supplementary file 1 [file ijerph-18-12077-s001.zip › ijerph-1427056-supplementary.pdf]

Supplementary:

Table S1. Values of all variables for each county in the BTH urban agglomeration

Prefecture	County	$PM_{2.5}$ (μg/m³)	GDP (billion yuan)	POP (thousand inhabitants)	LPI (unitless)	PLADJ (unitless)
Beijing	Changping	46.14	54.9502	1407.95	29.07	97.01
	Chaoyang	74.61	321.929	2752.21	91.74	99.10
	Daxing	77.48	112.313	1161.94	55.51	97.06
	Dongcheng	81.92	121.501	623.76	100.00	99.04
	Fangshan	51.97	39.3584	751.28	40.31	95.78
	Fengtai	78.57	81.2857	1575.82	94.12	98.79
	Haidian	62.51	358.602	2738.64	79.79	98.31
	Huairou	34.48	19.9513	313.37	48.17	94.62
	Mentougou	35.03	16.7576	320.01	44.32	94.43
	Miyun	41.63	18.6502	367.70	32.17	93.84
	Pinggu	53.59	17.0023	329.45	26.31	94.43
	Shijingshan	70.96	35.6595	485.59	100.00	98.79
	Shunyi	54.93	101.133	748.18	25.15	96.60
	Tongzhou	71.85	44.2232	934.53	24.37	96.69
	Xicheng	81.56	211.455	879.85	100.00	99.29
	Yanqing	30.51	8.58625	248.08	15.75	93.51
Baoding	Anguo	75.90	8.29972	314.02	13.22	95.27
	Anxin	78.63	4.16456	306.53	12.35	94.93
	Boye	76.42	3.12034	181.39	15.65	94.96
	Anxing	74.19	7.57805	413.59	12.43	95.15
	Dingzhou	73.11	21.9045	890.20	13.15	94.85
	Fuping	32.57	2.57703	172.80	5.50	88.40
	Gaobeidian	80.64	8.43167	396.86	18.12	94.82
	Gaoyang	79.74	6.83276	279.19	22.94	95.30
	Jingxiu	89.17	12.7953	332.18	75.32	97.19
	Laishui	39.98	5.07756	277.25	10.45	92.34
	Laiyuan	28.41	5.06077	211.61	16.78	91.86
	Li	77.11	6.02829	356.02	11.43	95.52
	Lianchi	92.49	24.6979	413.97	82.65	97.54
	Mancheng	64.25	5.99169	274.23	25.41	94.08
	Qingyuan	78.42	6.87146	507.03	6.66	95.53
	Quyang	53.44	5.06576	449.02	11.50	93.58
	Rongcheng	80.08	3.84675	193.79	10.14	95.15
	Shunping	57.92	3.95445	238.31	12.68	93.22
	Tang	48.20	4.91653	434.43	9.95	93.10
	Wangdu	75.00	3.8978	195.07	19.88	95.22
	Xiong	80.93	7.00524	286.32	16.00	94.91
	Xushui	74.41	7.286	447.08	17.25	95.07
	Yi	45.10	7.61654	400.68	10.42	92.24

	Zhuozhou	74.73	18.9385	496.59	24.50	94.16
Cangzhou	Cang	75.83	18.2257	548.51	4.31	93.80
	Dongguang	76.45	9.41416	260.01	20.42	93.10
	Haixing	70.05	3.71	183.82	11.07	93.00
	Hejian	79.30	18.8079	619.14	10.57	93.64
	Huanghua	73.41	20.7745	357.82	12.51	94.65
	Mengcun	71.94	5.40709	139.52	23.65	94.37
	Nanpi	75.28	6.41679	269.67	2.37	92.76
	Botou	76.11	13.3286	442.30	21.37	92.70
	Qing	79.54	11.9431	305.81	19.39	93.66
	Renqiu	80.63	39.2549	642.72	32.45	94.66
	Suning	75.76	9.02533	241.06	21.13	94.13
	Wuqiao	75.23	4.78772	183.10	23.09	91.26
	Xian	74.27	13.1825	450.71	8.91	93.66
	Xinhua	83.63	23.0602	561.69	87.67	97.69
	Yanshan	69.79	10.6554	373.29	12.41	93.03
	Yunhe	75.43	14.7802	230.41	69.97	96.63
Chengde	Chengde	30.29	8.332	291.46	8.55	88.79
	Fengning	19.41	7.3314	306.54	5.91	89.75
	Kuancheng	38.55	15.0677	201.45	5.70	88.61
	Longhua	22.94	8.23462	331.17	11.80	89.54
	Luanping	30.05	10.4403	222.31	9.62	89.53
	Pingquan	28.55	11.8421	366.84	13.49	88.98
	Shuangluan	32.24	7.48072	122.54	36.16	93.89
	Shuangqiao	34.27	11.3512	240.98	46.77	92.71
	Weichang	18.65	8.07357	414.77	6.40	87.37
	Xinglong	39.10	7.62174	242.43	18.51	88.70
	Yingshouyingzi	35.20	2.01126	41.68	71.45	95.55
Handan	Chengan	63.27	7.92046	284.17	14.24	94.30
	Chi	50.21	11.2622	320.86	14.19	93.76
	Congtai	70.25	17.3103	455.41	37.58	96.01
	Daming	69.49	9.30367	658.01	15.84	93.50
	Feixiang	66.30	6.13487	286.50	12.14	93.83
	Fengfeng	56.57	10.8016	316.52	40.85	94.55
	Fuxing	64.22	23.4424	417.77	56.91	96.60
	Guantao	69.34	6.73894	267.28	19.50	93.22
	Guangping	67.81	5.26222	222.91	22.98	94.59
	Hanshan	66.86	14.7344	376.08	22.11	95.55
	Jize	63.72	5.94093	227.69	16.91	94.10
	Linzhang	61.65	8.07397	522.28	14.68	93.76
	Qiuxian	69.16	4.78031	175.24	16.04	93.62
	Quzhou	66.10	7.69921	354.47	16.01	93.77
	Shexian	32.84	15.0164	293.34	39.97	93.04
	Wei	64.51	8.83943	688.01	9.51	93.88

	Wuan	41.87	39.7027	564.19	33.85	94.47
	Yongnian	64.63	14.7386	602.83	14.33	94.28
Hengshui	Anping	74.17	7.49002	233.76	26.75	94.75
	Fucheng	76.08	4.60993	247.66	12.58	92.37
	Gucheng	73.72	8.20475	392.37	14.35	93.21
	Yizhou	69.25	7.0195	273.87	18.63	93.31
	Jing	74.20	10.122	412.43	9.05	92.12
	Raoyang	74.26	4.29073	215.84	12.30	94.80
	Shenzhou	72.52	10.6792	424.10	11.91	94.43
	Taocheng	71.66	8.95457	297.00	50.94	95.91
	Wuqiang	71.50	3.3257	136.45	10.31	93.73
	Wuyi	73.50	4.72286	242.17	15.82	93.05
	Zaoqiang	72.72	5.83466	278.72	13.60	93.46
Langfang	Anci	82.21	13.6077	289.88	24.65	94.21
	Bazhou	83.22	23.9361	450.63	30.50	95.55
	Dachang	64.59	5.85939	86.65	24.16	93.99
	Dacheng	81.90	8.53661	383.94	7.31	92.91
	Guan	81.43	13.2519	372.39	17.04	93.77
	Guangyang	82.14	16.956	281.44	63.18	96.14
	Sanhe	64.01	35.5038	455.94	28.27	95.00
	Wenan	83.55	10.3784	391.80	25.02	94.69
	Xianghe	69.38	12.8679	233.27	25.20	94.20
	Yongqing	81.31	7.97599	296.31	8.42	92.91
Qinhuangdao	Beidaihe	50.32	4.41753	69.72	42.89	95.13
	Changli	56.02	14.3069	370.33	12.74	94.63
	Funing	47.30	16.0106	175.32	12.21	92.29
	Haigang	46.64	32.5136	595.76	45.79	96.35
	Lulong	51.59	8.67354	306.61	11.41	92.02
	Qinglong	40.89	8.87083	420.30	9.17	86.95
	Shanhaiguan	47.35	1.90409	59.74	72.15	95.70
Shijiazhuang	Gaoyi	68.59	5.98508	153.63	60.78	95.13
	Gaocheng	68.23	12.2692	582.41	21.92	95.26
	Jinzhou	70.99	18.2223	380.19	5.20	94.36
	Jingxing M	58.64	3.99285	62.46	66.54	96.23
	Jingxing	40.85	10.6642	257.00	23.61	92.42
	Lingshou	44.69	7.26599	252.42	19.68	92.74
	Luquan	60.71	6.41248	330.63	36.21	95.63
	Luancheng	68.19	3.15674	235.91	21.38	95.42
	Pingshan	34.19	13.3525	359.59	10.13	91.82
	Qiaoxi	41.21	29.7404	476.45	100.00	99.37
	Shenze	74.26	6.86853	169.61	25.19	94.75
	Wuji	68.83	12.4921	374.94	19.55	94.57
	Xinji	70.94	26.313	449.77	24.27	94.84
	Xinhua	83.63	23.0602	561.69	98.26	99.17

	Xinle	66.38	12.786	345.24	30.40	94.64
	Xingtang	51.40	9.35336	328.20	6.79	92.62
	Yuhua	82.98	14.7009	325.30	95.98	99.04
	Yuanshi	57.18	12.1139	300.42	13.56	94.81
	Zanhuang	46.56	6.55326	191.83	13.40	92.62
	Changan	85.52	28.2102	467.52	94.63	98.55
	Zhao	68.05	13.7373	415.59	18.74	94.38
	Zhengding	66.25	21.1322	391.92	29.11	95.26
Tangshan	Caofeidian	66.59	18.3629	234.56	44.11	99.00
	Fengnan	73.61	58.9266	411.76	18.71	95.61
	Fengrun	66.24	41.8831	580.75	23.70	94.72
	Guzhi	69.26	12.8765	222.92	64.51	96.67
	Pingkai	74.68	7.60981	155.88	59.57	96.68
	Laoting	59.74	22.2386	321.48	32.45	96.98
	Lubei	82.66	6.04824	138.94	94.07	98.68
	Lunan	78.85	17.9637	577.44	54.90	95.92
	Luannan	67.75	18.0146	320.67	37.04	95.71
	Luanzhou	62.18	32.6615	438.23	8.19	93.93
	Qianan	51.66	62.0508	536.92	17.48	95.81
	Qianxi	48.85	28.9623	296.08	14.67	92.30
	Yutian	66.79	26.5676	506.20	15.72	93.89
	Zunhua	54.67	36.2314	560.54	19.43	93.17
Xingtai	Baixiang	62.38	2.29243	137.11	22.74	94.24
	Guangzong	66.05	3.11757	238.74	16.28	94.56
	Julu	62.84	4.36619	303.43	15.37	94.34
	Lincheng	48.94	4.14026	154.85	7.37	91.74
	linxi	73.10	5.1717	278.12	15.12	92.85
	Longyao	62.72	6.1007	372.81	10.75	94.26
	Nangong	70.20	6.83829	346.08	19.56	94.09
	Nanhe	62.65	3.83684	262.55	19.11	94.16
	Neiqiu	46.86	4.89197	209.57	12.30	92.66
	Ningjin	65.33	12.8834	526.62	11.37	94.58
	Pingxiang	64.23	3.8024	245.16	15.58	94.27
	Qinghe	75.12	9.39708	313.93	29.92	94.34
	Renze	60.23	2.8673	240.89	18.07	94.38
	Shahe	49.56	15.3636	308.33	17.59	94.21
	Wei	70.61	5.23579	414.68	15.30	94.21
	Xiangdou	73.11	9.7578	309.44	56.32	96.69
	Xinhe	65.33	2.15843	127.81	33.51	94.42
	Xindu	40.84	18.1735	525.18	27.75	93.77
Zhangjiakou	Chicheng	19.58	5.90634	226.96	15.69	91.03
	Chongli	17.58	3.67408	113.70	7.74	88.09
	Guyuan	14.83	3.34123	169.02	22.19	92.11
	Huaian	21.95	4.4048	171.94	13.71	92.46

	Huailai	28.17	9.43237	266.44	20.76	92.87
	Kangbao	13.12	3.20582	200.82	9.61	91.21
	Qiaodong	24.84	5.57605	152.12	47.53	95.51
	Qiaoxi	41.21	29.7404	476.45	52.10	95.77
	Shangyi	14.94	2.50989	139.94	11.40	89.30
	Wanquan	21.04	5.85059	176.66	20.88	94.13
	Wei	26.66	5.57129	350.30	8.84	91.38
	Xiahuayuan	28.01	2.50404	72.90	32.68	92.83
	Xuanhua	24.67	13.2049	371.48	30.81	93.54
	Yangyuan	26.21	2.97554	194.74	13.39	92.20
	Zhangbei	14.55	6.79394	272.46	13.15	90.17
	Zhuolu	25.78	6.69324	256.45	6.95	90.08
Tianjin	Baodi	73.17	46.894	692.04	12.54	93.32
	Beichen	82.00	81.554	995.32	35.37	96.30
	Binhai New	74.33	603.553	1965.58	23.51	98.35
	Dongli	78.78	72.0392	777.55	52.80	97.16
	Dongping	82.99	10.6686	145.42	100.00	98.74
	Hebei	82.83	41.9806	655.96	100.00	98.96
	Hedong	82.25	54.2673	744.73	100.00	99.15
	Hexi	82.12	35.2006	434.26	100.00	99.30
	Hongqiao	83.77	19.4351	406.90	94.46	98.92
	Jizhou	59.34	70.6343	649.40	10.90	92.58
	Jinnan	74.97	53.2027	574.59	22.08	96.03
	Jinghai	79.18	6.78517	541.43	12.77	94.02
	Nankai	83.46	33.4422	460.56	100.00	99.19
	Ninghe	77.47	52.8932	383.63	16.33	93.32
	Wuqing	79.42	84.4421	994.02	19.33	93.91
	Xiqing	80.65	63.9924	529.19	73.37	97.58
